# Hand disease in scleroderma: a clinical correlate for chronic hand transplant rejection

**DOI:** 10.1186/2193-1801-2-577

**Published:** 2013-10-30

**Authors:** Kavit Amin, Bran Sivakumar, Alex Clarke, Anika Puri, Christopher Denton, Peter E Butler

**Affiliations:** Plastic and Reconstructive Surgery, Royal Free Hospital, Pond Street, NW3 London, UK

## Abstract

**Abstract:**

Chronic rejection remains a potential long-term consequence of hand composite tissue allotransplantation (CTA). Scleroderma has already been proposed as a model for chronic facial allograft rejection based on potential parallels of observed progression of disease and pathophysiology course. This study proposes a similar model for how chronic rejection may manifest itself in the context of hand CTA through the functional and psychological assessment of patients with scleroderma, should it occur.

**Methods:**

100 consecutive patients with a clinical diagnosis of scleroderma were recruited into the study. Subjective assessment of static hand disfigurement was carried out through the use of standardised digital photographs. Hand function was assessed through the measurement of active range of motion (AROM) and using the activities of daily living (ADL) and Disabilities of the Arm, Shoulder & Hand (DASH) questionnaire. Psychological and quality of life evaluation comprised the Hospital Anxiety Depression Scale (HADS) and the SF36 health survey.

**Results:**

Examination of standardised digital photographs of subjects revealed a variety of hand changes characteristic of scleroderma, ranging from mild to moderate through to severe. Objective assessment of hand disfigurement did not correlate with duration of disease, nor psychological distress. However, individuals with worsening disfigurement demonstrated poorer AROM. Longitudinally no deterioration in terms of function was seen over time in terms of the DASH and ADL results. Nevertheless deterioration of function did have a significant impact on quality of life. Overall HADS showed 22% of individuals as suffering from clinical levels of anxiety and 10% from clinical depression.

**Conclusion:**

Chronic rejection has not yet occurred in any of the hand transplants performed to date. Scleroderma results in a spectrum of chronic functional and psychological disability that provides a model for the potential outcome of chronic hand allograft rejection. Findings from this study provide insight into the impact of this progressive disease for patients and contribute to the information and consent process for patients considering hand composite tissue transplantation.

## Introduction

Composite tissue allotransplantation (CTA) is a significant development in reconstructive surgery. The world’s first hand transplant was performed in 1998 and was heralded as a major advance in reconstructive surgery (Dubernard et al. 1999). To date (August 2013), more than 85 hand transplants have been performed worldwide (IRHCTT, 2002).

Secondary to infection, the most significant complications encountered so far in hand CTA have been those of acute rejection (Unadkat et al. 2013). However, over the last 30 years significant advances in immunosuppression have been made with the development of potent immunosuppressive agents such as calcineurin inhibitors (cyclosporine, tacrolimus), anti-proliferatives such as mycophenolate mofetil, and corticosteroids. As a result of early recognition and treatment, acute rejection has become consistently reversible. Despite successful results, issues surrounding the risk-benefit ratio of long-term immunosuppression, and the potential for chronic rejection remain.

Currently, insufficient studies and analysis are available to define specific histopathological changes in chronic CTA rejection. Histological examination of one of the three hand transplants carried out in the United States has revealed features of ischaemic damage that may be the result of progressive arteriopathy of chronic rejection. In addition the onset of vascular narrowing, loss of skin adnexa, skin and muscle atrophy with fibrosis of deeper tissues has also been suggested (Pidwell & Burns 2007; Cendales et al. 2008). Clinicopathological features of graft rejection in the first human allograft at month 29 did not include deeper biopsies to assess the degree of deeper tissue rejection. This particular case of acute rejection was a result of non-compliance with immunosuppressive therapy. The authors report they did not observe scleroderma like features but suggest this most probably occurs with time (Kanitakis et al. 2003).

An erythematous maculopapular lesion present on skin allograft represents the macroscopic appearance of acute rejection. The main histological feature is that of monocuclear cell infiltrate, appearing first in the perivascular space of the dermis, progressing to the interface between dermis and epidermis. Necrosis then occurs resulting in dermal-epidermal separation. It is felt that the strong immune response can be attributed to increased antigenicity of skin. The high number of Antigen Presenting Cells and keratinocytes leads to expression of MHC molecules resulting in the secretion of chemokines, further attracting lymphocytes. This acute rejection response has been likened to that of atopic dermatitis and psoriasis, in that they mirror skin allograft rejection with regard to histopathological findings and composition of infiltrate in skin (Schneeberger et al. 2009).

Scleroderma has been proposed as a clinical correlate for chronic rejection in facial allograft transplantation, though to date this has not yet been proven as there have been no cases of chronic facial graft rejection (Sivakumar et al. 2010). Scleroderma is characterized by excessive extracellular matrix in the skin with atrophy of the epidermis. Histological analysis reveals perivascular invasion with interstitial lymphocytic infiltration, atrophy of adnexal structures, increased number of fibroblasts with thickening and intra luminal narrowing of the vasculature (Cendales et al. 2008).

Around 90% of patients report some deterioration in hand functionality (Casale et al. 1997). Patients present with swelling of the hands, skin thickening and arthralgias with a rapid symmetrical skin fibrosis affecting the distal extremities. Such impairment interferes with activities of daily living, more specifically with the performance of everyday tasks such as grasp, pinch and object manipulation (Sandqvist & Eklund 2000). In addition, the hands, like the face are visible to other individuals and therefore are potentially stigmatising (Heinberg et al. 2007).

This study has been designed to objectively and subjectively measure the functional and psychological impact of scleroderma on the hands of a cohort of patients with scleroderma, and attempt to understand the way in which chronic rejection may manifest in the context of rejection in chronic hand transplantation.

## Materials and methods

100 consecutive patients with a confirmed clinical diagnosis of scleroderma for consistency were recruited into the study. The diagnosis was based on the American College of Rheumatology classification criteria for scleroderma. Patients with localised scleroderma such as morphea or linear scleroderma were excluded from the study. Clinical diagnosis of scleroderma subtypes was confirmed via LeRoy’s criteria (1981). Ethical approval was gained from the Ethics Committee and full informed consent was obtained from each patient prior to recruitment into the study. The cosmetic, functional and psychological impact of the disease within hand scleroderma was assessed in the following ways.

### Objective measures

#### Observer rated disfigurement

**Figure 1:**
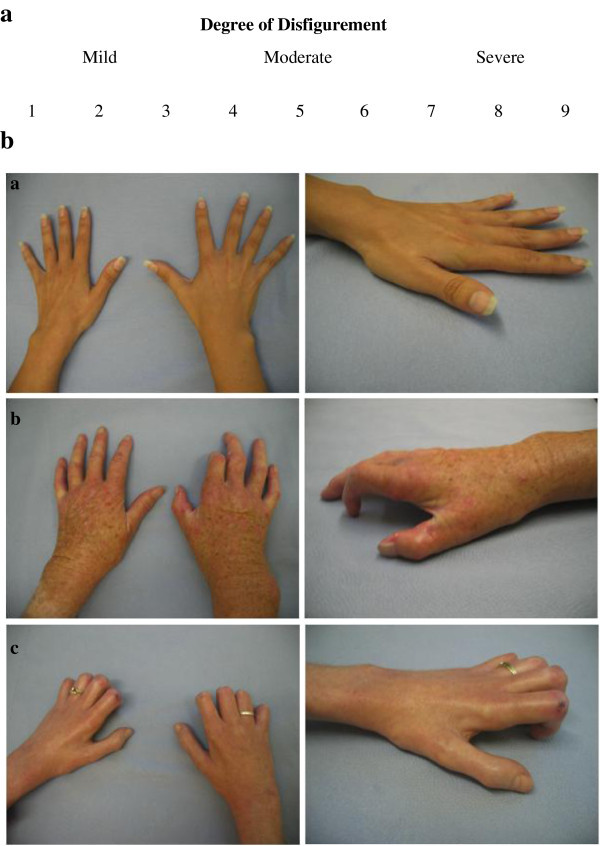
**Hand disfigurement grading and analysis. a**. Disfigurement was graded using a validated 9-point disfigurement scale. **b (a,b,c)**. Hand disfigurement analysis – standardized digital photographs of hands of scleroderma patients.

Standardised images of the palmar, dorsal and lateral views of both hands were captured using a digital Cannon Ixus 400 camera. Severity of disfigurement was ranked into 3 categories based on a 9-point disfigurement scale, ranging from 1–3 (mild disease), 4–6 (moderate disease) 7–9 (severe disease) (Figure 1a) (Katz et al. 2000). Three independent assessors rated the images in terms of whether they felt the appearance of patient hands represented mild, moderate or severe disease based on supplying a numerical number. Images with less than 100% agreement on category agreement were discarded.

#### Percentage active range of motion (AROM)

AROM measurements were taken at the wrist, metacarpophalangeal (MCP) and interphalangeal joints. The average percentage AROM of patients’ dominant hand joints was calculated by the same trained observer in line with the method employed for hand assessment by Lanzetta et al. in their study post hand transplantation (Provins 1997; Dubernard et al. 2003).

### Subjective measures

#### Activities of daily living (ADL) questionnaire

The activities of daily living (ADL) questionnaire was derived from studies of hand function as reported by the International registry on hand and composite tissue transplantation (Georges et al. 2004). Set up in May 2002, all groups performing hand transplants around the world have supplied information to the International Registry on Hand and Composite Tissue Transplantation (IRHCTT). The assessment allowed subjects to rank the ability to perform ADLs as, 0 (Impossible), 1 (Difficult) and 2 (Easy).

#### Disabilities of the Arm, shoulder & hand (DASH) questionnaire

The DASH questionnaire was developed by the American Academy of Orthopaedic Surgeons as a region-specific instrument for measuring upper-extremity disability and symptoms (Hudak et al. 1996). The questionnaire is validated and has been shown to be reliable in patients with various upper-extremity disorders. 88 patients completed the DASH questionnaire.

### Psychological and quality of life assessment

The following validated questionnaires were used:

#### The hospital anxiety and depression scale (HADS)

This is a psychometrically robust scale standardised on a hospital population. Each subscale (anxiety and depression) is scored from 0–21 with cut-offs of 8–10 (mild), 11–15 (moderate) and 16+ (severe) used as indicative of clinical caseness (Snaith & Zigmond 1986).

#### The short form-36 (SF-36)

The SF-36 comprises both a physical and mental health component and is widely used in clinical research. Previously used in scleroderma, scores have been shown to correlate well with severity of disease, and it has been reported to be accurate in evaluating QOL in this condition (Lewis & Wessely 1990; Georges et al. 2004; Danieli et al. 2005).

### Statistical analyses

Graph Pad Prism (Graph Pad Software, CA, USA) was used in the analysis of the data. A Bland-Altman analysis was carried out using Prism software, which showed no significant inter-observer variability with relation to the assessment of disfigurement. To compare two sets of variables, a Pearson correlation was used for normally distributed data, and a Spearman correlation for non-Gaussian data.

## Results

The demography of the cohort of 100 consecutive patients was typical of scleroderma with mean age of 54.7 years (range 17 to 100 years) and a female predominance (87.2%). Duration of disease (time since diagnosis) ranged from 1.5 years to 45 years, which was reflected in the broad range of disease patterns seen with a median of 8.5 years.

### Observer rated disfigurement

Digital photographs of 78 patients’ hands showed early skin changes including thickening, telangiectasia, a shiny taut appearance and soft tissue oedema (Figure 1b (a)). Areas of ulceration and pitted scars secondary to digital ischaemia together with muscular atrophy were seen in worsening cases (Figure 1b (b)). More advanced disease was compounded by joint contractures as a result of peri-articular fibrosis (Figure 1b (c)). Average disfigurement scores ranged from 1 to 8.67 with a median of 1.84. 51 (66%) patients were classified as mild, 17 (22%) patients as moderate and 9 (12%) patients as severe. Scores were not significantly associated with duration of disease (p > 0.05), nor with any of the QOL results.

### Percentage active range of motion (AROM)

**Figure 2:**
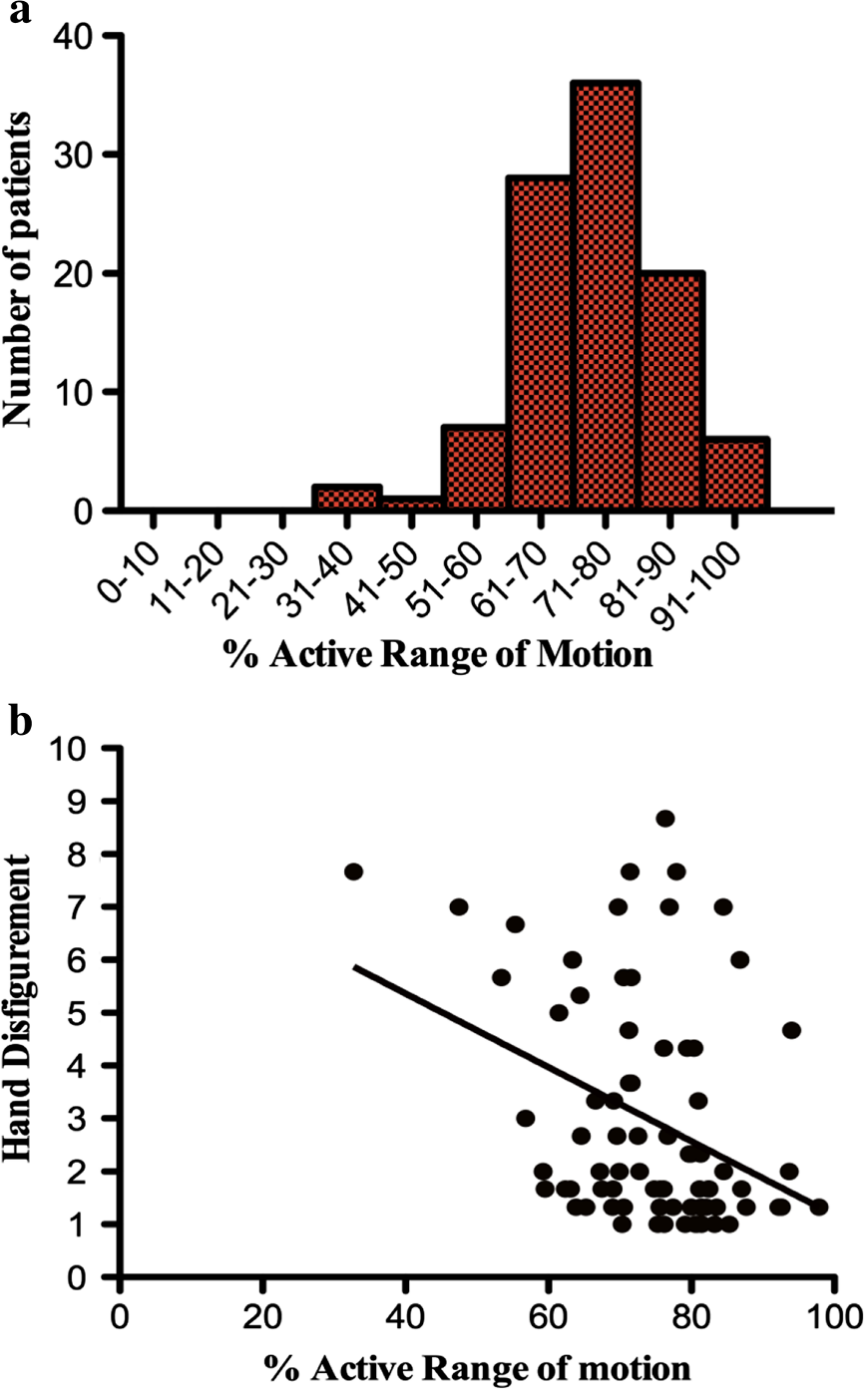
**Measure of function based on active range of movement. a**. Graph representing the percentage active range of movement within the cohort. **b**. Graph representing active range of movement against observer rated disfigurement.

Percentage active range of motion (AROM) was measured in the dominant hands of 98 patients. AROM scores ranged from 31% to 99% with a median of 75.5% (Figure 2a). Disease duration had no impact upon the active range of motion in our cohort (p > 0.05). Worsening AROM correlated significantly with worsening disfigurement (r = −0.359 p < 0.01) (Figure 2b). No significant correlation was seen between AROM and psychological distress. AROM did not correlate with our other functional assessments.

### Activities of daily living (ADL) questionnaire

100 patients completed the Activities of daily living (ADL) questionnaire. From a maximum score of 20, our scores ranged from 3 to 20, with a median score of 4. Ability to perform ADL was seen to not significantly deteriorate over time (r = −0.204, p < 0.01). A significant positive correlation between ability to perform ADLs, and the physical component of the SF-36 score was found (r =0.305 p <0.01). The QOL physical component of the SF36 declined with worsening ADL scores.

### Disabilities of the Arm, shoulder & hand (DASH) questionnaire

DASH scores from the study cohort ranged from a minimum of 1.79 to a maximum score of 89.17, with a median of 41.96. DASH scores significantly deteriorated over time (p > 0.05).

Upper limb disability reduced the ability to perform ADL (r = −0.537 p < 0.01) but did not correlate with hand disfigurement. Reduced QOL scores for both the physical (r = −0.535 p < 0.05) and mental component (r = −0.397 p < 0.05) were found with increasing upper limb disability.

### Psychological assessment

67% of patients completed the Hospital Anxiety and Depression scale (HADS) questionnaire (26). 40 (60%) patients were categorized as having non-case levels of anxiety, 12 (18%) as borderline anxiety and 15 (22%) of individuals categorized as experiencing clinical levels of anxiety. 68% of subjects classified as having non-case levels of depression, 15 (22%) as having borderline depression and 7 (10%) as having clinical depression.

69% of individuals completed the SF-36 health survey. Scores of the mental component ranged from 43.66 to 62.4, with a median of 48.9.

## Discussion

Chronic rejection remains a potential future consequence in hand CTA. Rejection amongst individuals having received a hand CTA are graded based on morphological features. There is agreement that progressive perivascular lymphocytic infiltrate occurs with increasing rejection with inflammation of the dermal stroma, epidermis and adnexa with epidermal apoptosis in severe cases. Scleroderma has been proposed as a clinical correlate for chronic rejection in facial allotransplantation based on potential parallels of pathophysiology and disease course (Sivakumar et al. 2010). Chronic changes in a nonhuman primate model of hand transplantation was characterized by arteritis, vasculopathy and intimal hyperplasia progressing to vessel occlusion. The grafts appeared pale and oedematous, these findings mirroring to a degree findings in progressive scleroderma (Mundinger et al. 2013). Previous studies have shown that skin deformity is a core stressor within the scleroderma population (Benrud-Larson et al. 2002).

Assessment of standardised photographs revealed a broad range of disease, from mild through to severe. The cohort was representative of the spectrum of changes that would be seen in a chronic rejection process. Presentations in 4 chronic hand rejection transplant recipients have involved a desquamative red papular rash, lichenification of the palm and dystrophic changes to the nail with eventual loss of the nail, similar charateristics seen in our scleroderma population (Ravindra et al. 2012).

In our study, with time appearance did not worsen. Furthermore, despite many subjects reporting skin changes as a major concern, no relationship could be seen between psychological wellbeing and severity of disfigurement. This implies that appearance alone is not a defining factor in patients’ perception of the severity of hand disease. However, this may in some way be related to the cross-sectional nature of our study. A longitudinal analysis of a larger cohort of patients would be useful (van Lankveld et al. 2007).

Individuals with scleroderma commonly suffer from rheumatic problems. Articular manifestations may be the presenting feature in the majority of patients, and clinically manifest as arthralgia, flexion contractures and in fewer cases as arthritis which have an impact on functionality (Pope 2003). In line with our cosmesis findings, disease duration appeared to have no effect on hand joint mobility. However, with increasing disfigurement, we saw worsening joint motion. Additionally, we identified that with longer disease duration, the more limited individuals were in their ADL. However, despite deterioration in mobility, patients were able to adapt remarkably well in managing their activities of daily living. Similar results have been found in other studies with chronic hand pathologies (Van Zelst et al. 2006). This most likely reflects the chronic deterioration in hand mobility, which enables adaptation. This may explain why psychological scores were not affected by decreasing range of motion. As we would expect, increases in ADL brought about a greater physical feeling of QOL.

The DASH was primarily used in the study to identify whether its use had similar outcomes to the International Registry on hand transplantation ADL questionnaire. The registry outcomes have never been applied to the scleroderma population before, and in demonstrating the correlation with SF-36 and the DASH, similarities between the two populations have been demonstrated which support the proposal that scleroderma is a useful clinical model for hand CTA rejection. People with higher scores on the DASH representing greater upper limb disability, were found to demonstrate poorer QOL outcomes with lower scores on both the physical and mental components of the SF-36.

Scleroderma affects hand function and appearance, which can lead to psychological distress. Psychological problems are common amongst patients with chronic illnesses such as scleroderma and may interact and exacerbate functional disability and global disease. 40% of individuals were borderline or classified as clinical cases with anxiety. Furthermore, 32% of the cohort either had borderline depression or were clinical cases. Despite the majority of participants falling within the non-case category, significant psychological distress was evident in a small proportion of the cohort. In our cohort, pain was a powerful predictor of depression and increasing feelings of subjective limb disability, therefore we would suggest that adequate pain control in these individuals requires careful consideration (Benrud-Larson et al. 2002).

It is striking that individuals with scleroderma appear to adapt their behaviour to cope with functional changes, such that levels of disability remain remarkably low. Many of the problems the condition poses are manageable, and psychological resilience appears high overall with a minority of patients exhibiting psychological distress that require clinical intervention. Nonetheless, ongoing and regular screening for both pain and psychological distress would be pertinent if chronic graft rejection were to take place, allowing for considerable scope for positive early intervention. Experience from solid organs transplantation shows that high numbers of acute rejection results in a higher incidence of chronic rejection. Similarly, in a rat hind limb model, nerve regeneration and muscle activity were shown not to be affected by a small number of acute rejections. However, with increasing acute rejection, there is a significant decline in function leading to atrophic and fibrotic muscle. There was evidence of mononuclear cellular infiltration (Unadkat et al. 2013). It has been suggested that the low rate of rejection observed in current hand allograft is attributed to the short follow-up and early recognition allowing reversal of acute rejection. Additional evaluation of acute and chronic rejection in human hand and face allograft is required to define its characteristics since no definite signs of chronic rejection are present in any allografts to date, including the longest surviving allograft of 12 years (Whitaker et al. 2008).

It may be that the connection between scleroderma and chronic rejection is not as strong as proposed within the model. The pathophysiological processes do somewhat differ, in that scleroderma manifests with thickening of connective tissue within the deep dermis, subcutaneous fat and muscular fascia, with thinning of tissues in chronic rejection. An important difference between those from the scleroderma and hand transplant cohort resides in the notion that hand amputees are healthy and selected for transplantation on the basis they do not have significant comorbidities impacting on infection and malignancy. In addition, transplantation of the hand is not life-saving and does not maintain long-term survival.

## Conclusion

We believe using scleroderma as a model for chronic rejection to be highly informative since it represents a spectrum of chronic functional and psychological disability that may be encountered in chronic hand allograft rejection, and therefore provides a useful addition to the patient information and consent process. Cosmesis was found not to be a significant stressor, and does not appear to impact significantly on QOL. However, functional deterioration is considered paramount but looking overall at individual QOL, most patients appear to be coping well. After hand CTA we would hope for a dramatic increase in function, health and well-being. The gradual deterioration in function from a chronic rejection process may have a psychological impact on transplant recipients, but we have shown that individuals are able to cope with this.
